# *BRCA2* reversion mutation confers resistance to olaparib in breast cancer

**DOI:** 10.1002/ccr3.7537

**Published:** 2023-06-23

**Authors:** Shinya Yamamoto, Kei Kawashima, Yoshie Fujiwara, Shoko Adachi, Kazutaka Narui, Chiaki Hosaka, Rina Takahashi, Sho Tsuyuki, Makoto Sugimori, Miki Tanoshima, Mahato Sasamoto, Masanori Oshi, Akimitsu Yamada, Chikara Kunisaki, Itaru Endo

**Affiliations:** ^1^ Department of Breast and Thyroid Surgery Yokohama City University Medical Center Yokohama Japan; ^2^ Department of Clinical Genetics Yokohama City University Medical Center Yokohama Japan; ^3^ Department of Cancer Genome Medicine Yokohama City University Medical Center Yokohama Japan; ^4^ Department of Gastroenterological Surgery Yokohama City University Graduate School of Medicine Yokohama Japan

**Keywords:** BRCA, breast neoplasms, genomics, olaparib

## Abstract

**Key Clinical Message:**

A rare missense mutation was identified as a reversion mutation using cancer genomic profiling and a suspected mechanism underlying resistance to olaparib in breast cancer.

**Abstract:**

A 34‐year‐old woman with breast cancer and *BRCA2*: p.Gln3047Ter was treated with olaparib. After tumor progression, cancer genomic profiling testing using liquid biopsy revealed *BRCA2* p.Gln3047Ter and p.Gln3047Tyr, with 48.9% and 0.37% allele frequency, respectively. These findings shed light on reversion mutation as a mechanism of resistance to olaparib in breast cancer.

## Graphical abstract

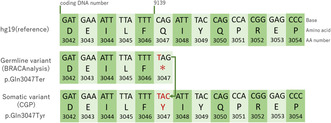

## 1 INTRODUCTION

The protein‐encoded *BRCA* is involved in homologous recombination repair and plays an important role in DNA double‐strand break repair. Poly (adenosine diphosphate ribose) polymerase (PARP) plays a vital role in DNA single‐strand break repair.^1^ Therefore, olaparib, a PARP inhibitor, is effective for tumors with a germline *BRCA* mutation owing to its synthetic lethality.^2^ The OlympiAD trial demonstrated the efficacy of olaparib in patients with human epidermal growth factor receptor type 2 (HER2)‐negative metastatic breast cancer with a germline *BRCA* mutation.^2^ The median progression‐free survival was significantly longer in the olaparib group than in chemotherapy of the physician's choice group (7.0 vs. 4.2 months; *p* < 0.001).^2^

In recent years, several mechanisms of resistance to PARP inhibitors have been proposed,^3^ including protein alteration in the homologous recombination pathway, altered expression of a protein in the DNA replication fork protection pathway,^4^ epigenetic modifications, restoration of ADP‐ribosylation, and pharmacological alterations.^3^ Importantly, a reversion mutation has been recently reported as a mechanism of resistance to the PARP inhibitor olaparib.^5^, ^6^

Herein, we describe a case of a patient with breast cancer with a *BRCA2* pathogenic variant that was resistant to olaparib and was suspected to be a reversion mutation based on cancer genomic profiling.

## 2 CASE PRESENTATION

**FIGURE 1:**
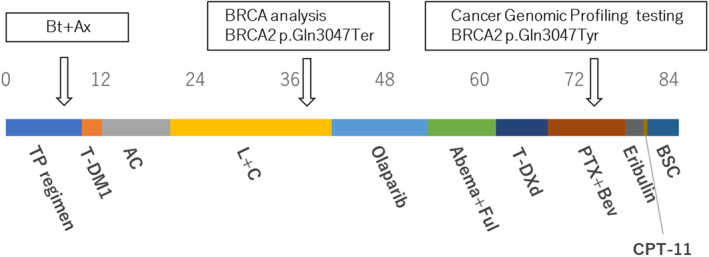
Timeline of the treatment course. AC, adriamycin and cyclophosphamide; Abema+Ful, abemaciclib and fulvestrant; BSC, best supportive care; Bt+Ax, total mastectomy and axillary lymph node dissection; CPT‐11, irinotecan; L+C, lapatinib and capecitabin; PTX + Bev, bevacizumab and paclitaxel; TP, trastuzumab and pertuzumab; T‐DM1, trastuzumab emtansine; T‐Dxd, trastuzumab deruxtecan.

**FIGURE 2:**
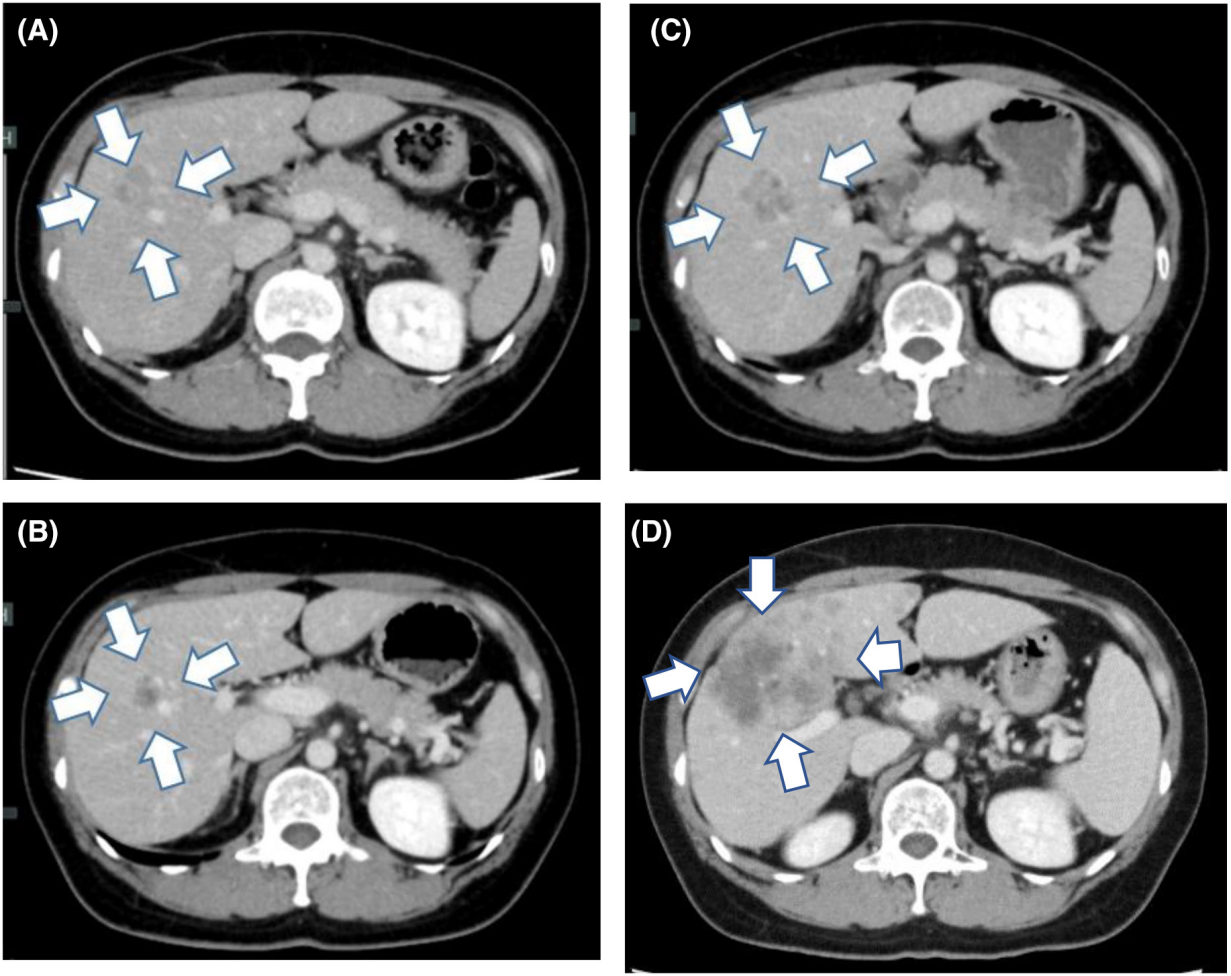
Liver metastasis on computed tomography. (A) Liver metastasis before olaparib administration (arrows). (B) Liver metastasis has shrunk (arrows). (C) Liver metastasis has progressed after 12 months of olaparib administration (arrows). (D) Liver metastasis (arrows) at the time of cancer genomic profiling testing.

**FIGURE 3:**
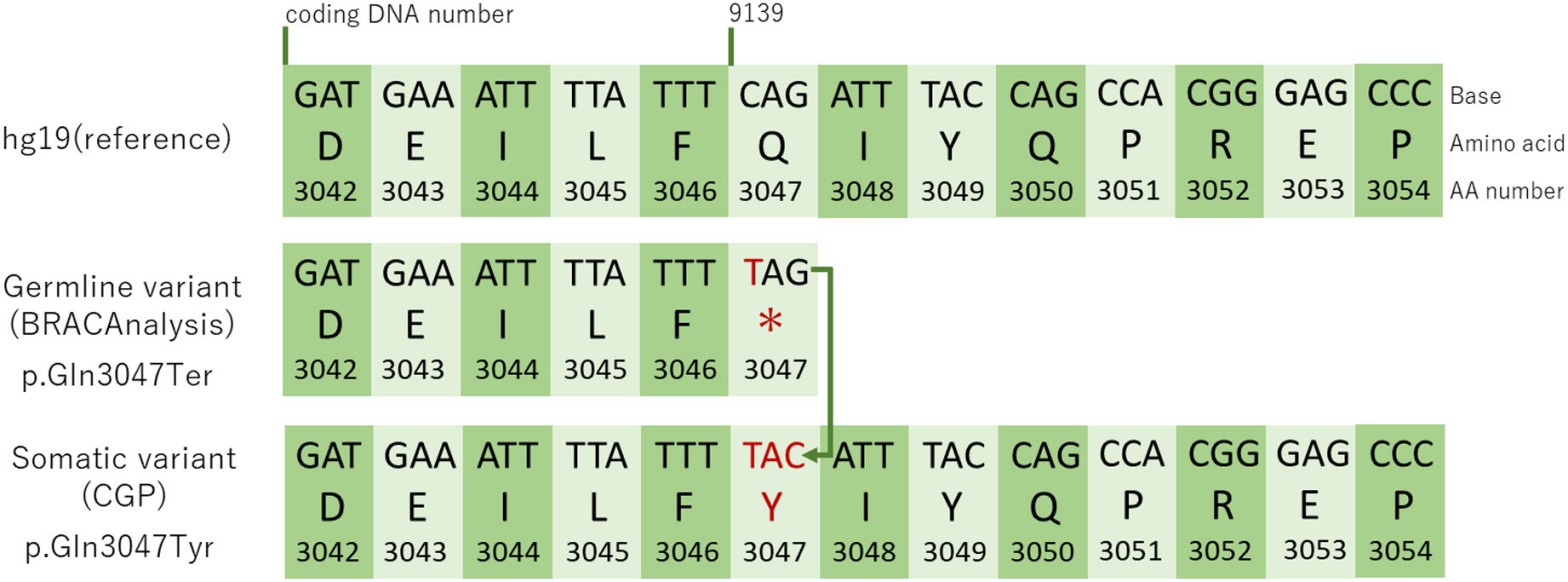
Schema of the *BRCA2* mutation. Middle row: result of BRCA genetic testing. The following pathogenic variant in *BRCA2* is detected: p.Gln3047Ter. Lower row: result of cancer genomic profiling testing. The following new mutation is identified: p.Gln3047Tyr. CGP, cancer genomic profiling testing.

A 34‐year‐old woman presented to our hospital with a mass in her right breast, which she noticed after giving birth to her second child. Breast cancer was diagnosed via core needle biopsy. The tumor was classified as invasive ductal carcinoma, estrogen receptor (ER) positive, progesterone receptor (PgR) positive, and HER2 positive. The patient's family history was as follows: her maternal grandmother suffered from stomach cancer in her seventies, her mother suffered from gallbladder cancer in her fifties, and her maternal aunt suffered from stomach cancer in her thirties. The timeline of the treatment course is shown in Figure 1. The patient received eight courses of docetaxel–pertuzumab–trastuzumab therapy (75 mg/m^2^ docetaxel on Day 1, 420 mg/kg pertuzumab on Day 1, and 6 mg/kg trastuzumab on Day 1 every 3 weeks). Subsequently, right total mastectomy and axillary lymph node dissection were performed. The main pathological findings were a residual tumor diameter measuring 53 mm, a number of residual lymph node metastases found in four of 18 nodes, ER and PgR positivity, HER2 negativity, and a histological response to preoperative therapy of grade 1a. Postoperatively, the patient received chemotherapy and endocrine therapy (pertuzumab–trastuzumab, tamoxifen, and a luteinizing hormone‐releasing hormone agonist). Magnetic resonance imaging revealed liver metastases. After three courses of trastuzumab emtansine (3.6 mg/kg every 3 weeks), the metastatic lesions had progressed. Subsequently, adriamycin and cyclophosphamide (AC) therapy (60 mg/m^2^ adriamycin on Day 1 and 600 mg/m^2^ cyclophosphamide on Day 1 every 3 weeks) was administered. Considering cardiotoxicity, AC therapy was completed after 11 courses (total adriamycin dose: 495 mg/m^2^). Thereafter, lapatinib (1250 mg/kg daily) plus capecitabin (3600 mg/kg on Days 1–14 every 3 weeks) therapy was administered. After 20 months, the metastatic tumors showed progression (Figure 2A). *BRCA* genetic testing (BRACAnalysis® [Myriad Genetics]) was conducted using the patient's blood sample as the germline sample, and a pathogenic variant was detected. The variant using hg19 as the reference genome was *BRCA2*: c.9139C>T (p.Gln3047Ter) (Figure 3). Considering her age, the BRCA test should have been done earlier, but considering the timing of insurance coverage in our country, it was decided at this time. As the results of immunostaining of the surgical specimen were negative for HER2, olaparib (600 mg/kg daily) was administered. The tumor size reduced after olaparib administration (Figure 2B); however, progression of liver metastases was noted after 12 months of olaparib administration (Figure 2C). Repeated biopsy of the liver metastasis revealed an ER‐positive, PgR‐positive, and HER2‐negative tumor. Abemaciclib (150 mg twice daily) plus fulvestrant (500 mg every 4 weeks) therapy was administered. After 8 months, metastatic lesions progressed. Considering the possibility that HER2‐positive cells were dominant, trastuzumab deruxtecan (5.4 mg/kg every 3 weeks) was administered. After 4 months, metastatic lesions progressed. The HER2 status was not confirmed, but we decided to use drugs that had not been previously used; therefore, bevacizumab (10 mg/kg on Days 1 and 15 every 4 weeks) plus paclitaxel therapy (90 mg/m^2^ on Days 1, 8, and 15 every 4 weeks) was administered. Cancer genomic profiling (FoundationOne® Liquid CDx) was performed during bevacizumab plus paclitaxel therapy. The sample at the time of surgery was >3 years old, and the amount of specimen at the time of liver biopsy was insufficient; therefore, liquid biopsy was performed. Figure 2D shows computed tomography images of liver metastasis when cancer genomic profiling testing was performed. The variants detected were *BRCA2* c.9139C>T (p.Gln3047Ter) and *BRCA2* c.9139_9141delinsTAC (p.Gln3047Tyr) mutations, with 48.9% and 0.37% allele frequencies, respectively (Figure 3). The other variants that were detected were as follows: DNMT3A c.2183G>A (p.G728D) with 0.6% allele frequency, LTK c.548C>T (p.S183F) with 45.2% allele frequency, LTK c.2120G>A (p.W707Ter) with 48.4% allele frequency, MSH2 c.2197G>A (p.A733T) with 48.0% allele frequency, RB1 c.2455C>G (p.L819V) with 46.0% allele frequency, SPOP c.931C>T (p.L311F) with 46.0% allele frequency, and MYC rearrangement (chr8:128748880‐chr14:26292352). After 9 months of bevacizumab plus paclitaxel therapy, metastatic lesions progressed. After that, we decided to use drugs that had not been used previously; therefore, eribulin was sequentially administered (1.1 mg/m^2^ on Days 1 and 8 every 3 weeks). However, after 3 months, metastatic lesions progressed; thus, irinotecan (100 mg/m^2^ on Days 1, 8, and 15 every 5 weeks) was administered. After one course, the patient's general condition deteriorated, and the policy of best supportive care was adopted; 4 months later, the patient died.

## 3 DISCUSSION

A reversion mutation is a well‐discussed mechanism of resistance to PARP inhibitors. Waks et al.^4^ reported a reversion mutation as the most commonly observed mechanism of resistance to PARP inhibitors or platinum chemotherapy in eight patients with metastatic breast cancer with *BRCA1/2* mutations. Tobalina et al.^7^ reported the occurrence of reversion mutations in 26.0% (86/327) patients with *BRCA1 or BRCA2* mutations, including 27 patients with breast cancer, with a higher percentage in *BRCA2* compared with *BRCA1* (30.7% vs. 22.0%).^7^ Lai et al.^8^ evaluated *BRCA* reversion mutations in a study of homologous recombination deficiencies. In their study, 157 reversion mutations among 5574 samples were identified, and most reversion mutations were in exon 11 for both *BRCA1* and *BRCA2*, which was the largest exon for both genes. Pettitt et al.^9^ analyzed and reported 308 homologous recombination gene reversions associated with a PARP inhibitor or platinum resistance. In *BRCA1*, the reversion mutations occurred throughout the *BRCA1* coding sequence. However, in *BRCA2*, there were “hotspots” and “deserts.” The frequency of reversions 3′ to coding sequence position 7617 (exon 16 onward) was significantly lower than the expected frequency based on the incidence dataset. However, numerous reversion mutations in the N‐terminal c.750–775 regions have occurred. Therefore, our presented case might be relatively rare. However, as the authors' indicated, PARP inhibitors are routinely used in clinics and some reversion mutations will no longer be considered novel enough to be reported. Therefore, reversion mutations in this area may be underestimated.

Weigelt et al.^10^ reported the detection of reversion mutations using circulating cell‐free DNA in breast and ovarian cancers. Tumor tissue was not synchronously collected; therefore, it was not possible to validate the presence of the putative reversion mutations in the tumor, which was a limitation in Weigelt et al.'s study.^10^ Similarly, the reversion mutation was not confirmed using tissue biopsy in our case herein. A repeat tumor biopsy from liver metastases and liquid biopsy to confirm the amplification of newly detected secondary variants were planned. However, these were not performed due to the deterioration of the patient's condition and the fact that no additional treatment would be administered.

In addition, there was a concern in our case. The occurrence of true reversions to the wild type was previously reported^9^; however, true reversions are challenging to identify using liquid biopsy alone. Thus, the prevalence of true reversions may be underestimated, and it is possible that our case also had a true reversion. An allele frequency of 0.37% is relatively low, and the mutation was considered subclonal. The resistance mechanism might not be caused by a reversion mutation only.

There are several reports on the treatment after PARP inhibitor resistance. Reversions have been predicted to encode immunogenic neopeptides; thus, immunotherapies may also be an option for direct targeting of the revertant protein.^9^ As another strategy for acquired resistance to PARP inhibitors, a cyclin‐dependent kinase 12 inhibitor^11^ or WEE1 kinase inhibitor^12^ can be used. To prove the efficacy of these treatments, it is important to continue to investigate PARP inhibitor resistance cases.

## 4 CONCLUSION

We experienced a case in which the possibility of a reversion mutation was suggested as a mechanism of resistance to olaparib. The amount of research into these cases has so far been insufficient to elucidate the mechanism of resistance to olaparib; therefore, reporting each case is of paramount importance.

## AUTHOR CONTRIBUTIONS

**Shinya Yamamoto:** Conceptualization; data curation; investigation; writing – original draft; writing – review and editing. **Kei Kawashima:** Data curation; writing – review and editing. **Yoshie Fujiwara:** Data curation; writing – review and editing. **Shoko Adachi:** Data curation; writing – review and editing. **Kazutaka Narui:** Data curation; writing – review and editing. **Chiaki Hosaka:** Data curation; visualization; writing – original draft; writing – review and editing. **Rina Takahashi:** Data curation. **Sho Tsuyuki:** Data curation; writing – review and editing. **Makoto Sugimori:** Data curation; writing – review and editing. **Miki Tanoshima:** Data curation; writing – review and editing. **Mahato Sasamoto:** Data curation; writing – review and editing. **Masanori Oshi:** Data curation; writing – review and editing. **Akimitsu Yamada:** Data curation; writing – review and editing. **Chikara Kunisaki:** Writing – review and editing. **Itaru Endo:** Writing – review and editing.

## FUNDING INFORMATION

None.

## CONFLICT OF INTEREST STATEMENT

The authors declare that there are no conflicts of interest regarding the publication of this article.

## ETHICS APPROVAL

This study was conducted ethically in accordance with the World Medical Association Declaration of Helsinki.

## CONSENT

Written informed consent was obtained from the patient to publish this report in accordance with the journal's patient consent policy.

## Data Availability

Data sharing is not applicable to this article as no datasets were generated or analyzed during the current study.
